# Analysis of Long-Term Outcomes Following Surgical Contracture Release of the Elbow: A Case Series

**DOI:** 10.7759/cureus.14691

**Published:** 2021-04-26

**Authors:** Brittany M Ammerman, Gary Updegrove, Padmavathi Ponnuru, April Armstrong

**Affiliations:** 1 Orthopaedics, Penn State Health Milton S. Hershey Medical Center, Hershey, USA

**Keywords:** elbow contracture, elbow stiffness, contracture release, elbow surgery, contracture

## Abstract

Background

Elbow contracture is a debilitating condition with an incidence ranging from as low as almost 1% to as high as 20% and results in significant limiting consequences on a patient’s activities of daily living (ADLs). Postoperative rehabilitation is important in maintaining the range of motion and sustaining an improved range of motion. The purpose of this study was to evaluate the long-term results of elbow contracture release surgery and the effect of an occupational therapy/physical therapy (OT/PT)-guided, self-directed rehabilitation program following surgery, without the use of continuous passive motion (CPM) devices.

Methods

We enrolled patients who had undergone elbow contracture release surgery from 2005 to 2016 at a single institution under the senior author. The evaluation included objective measurements of range-of-motion, strength, and neurological sensory testing. Provocative testing of the elbow and hand was performed. American Shoulder and Elbow Surgeons-elbow (ASES-e), Simple Shoulder Test-elbow (SST-e), Disabilities of the Arm and Shoulder (DASH), Mayo Elbow Performance Index (MEPI), Short Form-36 (SF-36), and an investigator questionnaire were completed.

Results

We enrolled 19 patients, six female and 13 male, with an average follow-up of 58.9 months (SD± 39.8, Range 22-117). We showed improvement and sustained motion between preoperative and postoperative research visit flexion (p<0.001) and flexion extension-arc (p<0.01). The mean increase in flexion was 98° to 131° and the flexion-extension arc was 36°. Patients were satisfied with the decision to undergo surgery and had sustained ability to complete ADLs.

Discussion

This patient cohort demonstrated a statistically significant increase, as well as long-term maintenance in the flexion and flexion-extension arc. A self-directed, OT/PT-guided, therapy program without CPM was effective. Patients showed good outcomes and were satisfied with their ability to perform ADLs, decreased pain, and the decision to undergo surgery.

## Introduction

Elbow contracture is a debilitating condition with significant limiting consequences on a patient’s activities of daily living (ADLs) [1]. Due to the elbow joint’s distinct anatomy, particularly the presence of three separate articulations within a single synovial cavity, the abundance of soft tissue providing joint stability, and the proximity of the brachialis to the anterior capsule, the joint is susceptible to stiffness and contracture [2]. The range of motion (ROM) and functioning of the elbow allow critical positioning of the hand in space and the performance of ADLs. The physiologic full ROM of the elbow is 0° of extension to 140°-150° of flexion, 80°-85° of supination, 75°-80° of pronation [3]. A functional ROM for ADLs has been shown to be 100° for both the flexion-extension arc (30°-130°) and the pronation-supination arc (50° in either direction) [4]. However, this suggested functional ROM has been recently brought into question, as contemporary ADLs, such as those associated with cell phone use and computer tasks, may require increased ROM [5-7]. Loss of ROM in the elbow joint has been defined as loss of extension greater than 30° or flexion of less than 120°, though it is important to note functional limitations can be appreciated with a less severe ROM loss [8]. Due to the debilitating nature of elbow contracture, the goals of treatment include restoring a more functional arc of motion, decreasing or eliminating pain, and providing joint stability [9]. While some patients can be successfully managed with nonoperative treatment, such as physical therapy, which may or may not include the use of dynamic or static splinting, some cases are refractory to conservative treatment and surgery is indicated [9].

Both open and arthroscopic surgical contracture release techniques have demonstrated the ability to restore motion to a stiff elbow [1-2,8-16], however, the optimal postoperative rehabilitation protocol following surgical contracture release remains unclear [10]. Some authors recommend continuous passive motion (CPM) after surgical contracture release [11-15] to begin elbow ROM immediately postoperatively, but there is limited scientific evidence to support the use of CPM [14,16]. There are also disadvantages of CPM use to consider, including the bulky nature of the CPM device and increased costs to the patient.

The purpose of this study is to evaluate the long-term functional results of elbow contracture release surgery, in addition to the effect of occupational therapy (OT) or physical therapy (PT)-guided self-directed rehabilitation program following surgery, without the use of continuous passive motion (CPM) devices. We hypothesize that patients maintain improvement in ROM when using this non-CPM rehabilitation approach.

## Materials and methods

This study’s protocol was approved by the Institutional Review Board (IRB # PRAMS 039744EP). The orthopedic surgical database at our institution was queried for patients who had undergone elbow contracture release surgery, open or arthroscopic, by the senior author, from June 2005 to December 2016. This was a single-center study. Patients who met eligibility requirements were contacted by the research associate via telephone. Inclusion criteria were skeletally mature subjects 18 years of age or older who underwent open or arthroscopic contracture release performed by the senior author between June 1, 2005, and March 30, 2017. Exclusion criteria were less than 18 years old and inability to provide informed consent. Informed consent was obtained, and a research-only visit was scheduled with the research associates.

The research visit evaluation was conducted by the research associate and medical student for all patients and included objective measurements of ROM, strength, and neurological sensory testing. ROM measurements included: elbow flexion, extension, pronation, and supination using standard universal goniometer measurements. Grip strength was measured using a hand dynamometer. Other physical examinations included an assessment of Tinel’s sign at the elbow, cubital tunnel stretch test, ulnar nerve subluxation, and the 2-point discrimination test [17-21]. Threshold sensitivity testing included the Semmes-Weinstein monofilament test [22]. Each patient completed a Hand Pain and Numbness Diagram [23] (Appendix A). Subjective outcome measures were then completed by the patient, including American Shoulder and Elbow Surgeons-elbow (ASES-e) [24], Simple Shoulder Test- elbow (SST-e) [25], Disabilities of the Arm and Shoulder (DASH) [26], Mayo Elbow Performance Index (MEPI) [27], Short Form-36 (SF-36) [28], as well as an investigator-written questionnaire (Appendix B). The ASES-e was scored by subscale (pain, function, and surgery satisfaction), with the ASES pain score ranging from 0-50 points, with 50 points indicating no pain; function score ranging from 0-36 points, with 36 points being full functionality; and surgery satisfaction ranging from 0-10 points, 10 being full satisfaction. The SST-e clinical outcome score assessed the patient’s ability to perform ADLs and was scored on a scale of 0-100, with 0 being unable to perform and 100 being able to easily perform, The DASH assessed functionality, pain, and other symptoms and was scored on a scale of 0-100, with 0 equating no disability and 100 being most severe disability. The MEPI measured elbow function across four domains: pain, stability, motion, and daily functional tasks and was scored on a scale of 0-100. MEPI scores are categorized as excellent (90-100), good (75-89), fair (60-74), and poor (0-59). The SF 36 score reflects the physical function score and was scaled 0-100, with 100 being a greater health-related quality of life.

The postoperative rehabilitation protocol was standardized for all 19 patients. In the immediate postoperative period, the patient was immobilized in a padded splint with their arm in full extension and elevated overnight via the intravenous (IV) pole. Patients who had an outpatient arthroscopic procedure were still splinted in full extension and asked to keep their arm elevated on pillows overnight. The splint was removed on postoperative Day 1 and active-assisted ROM in flexion, extension, pronation, and supination were started under instruction by occupational therapy. For inpatients, the patients were instructed to continue with their ROM exercises every waking hour on hour until they were able to see their outpatient therapist. For outpatients, they were scheduled to see the therapist the following day after surgery for splint removal and to start exercises. Patients were also provided with a nighttime extension splint. A sling was provided for resting comfort only during the day and patients were advised to wean from sling use by two weeks postoperative or based on their comfort level. Patients attended regular sessions for occupational or physical therapy to continue with stretching. Patients were provided a daily home exercise program (HEP) with emphasis on the terminal end ROM by OT/PT and were advised to complete the HEP multiple times daily. Patients continued with their OT/PT session and HEP until independence with ADLs was achieved and full ROM was achieved or ROM progress had plateaued.

Data are represented as mean ± standard deviation (SD) for each group and a comparison of the groups was done using the paired t-test (two-tailed). Statistical analysis of the data was performed using GraphPad Prism8 (GraphPad Software, San Diego, CA).

## Results

We were able to enroll 19 patients in the study: six female and 13 male. The mean age of the study population at the time of elbow contracture release surgery was 48.6 (SD ± 13.2, range 19-71). Time from surgery to final clinical postoperative follow-up averaged 8.1 months (SD ± 6.3, range 2-21). The average time of follow-up from surgical date to research visit was 58.9 months (SD± 39.8, range 22-117). Further demographic and descriptive data are reported in Table 1.

**Table 1:** Patient characteristics

Characteristic	Mean ± SD or Number of Patients (%)
	(n=19)
Age	48.6 ± 13.2
Sex	
Male	13 (68.4)
Female	6 (31.6)
Laterality	
Left	9 (47.4)
Right	10 (52.6)
Handedness	
Dominant	9 (47.4)
Non-Dominant	10 (52.6)
Heterotopic Ossification	6 (31.6)
Prior Surgery for Initial Injury	11 (57.9)
Surgical Approach	
Open	15 (79.9)
Arthroscopic	4 (21.1)
Ulnar Nerve Transposed	12 (63.2)

Of the 19 patients, 15 underwent an open contracture release and four an arthroscopic contracture release. Operative reports provided details about the surgical approach and exposure for both open and arthroscopic contracture release procedures. Of the 15 open elbow contracture release surgeries, 14 were done through a posterior midline incision, the other through a medial incision. Regarding the deep exposure for surgical release, three were performed via the medial approach only, four via the lateral approach only, and eight via both the medial and lateral approaches. Twelve patients had an ulnar nerve transposition at the time of elbow contracture surgery, with one being a revision. Indication for transposition included scarring, compression, or mobilization of a nerve to allow for the release of the posterior bundle of the medial collateral ligament to increase ROM. Table 2 further details the surgical approach for each of the 15 patients who underwent an open contracture release.

**Table 2:** Elbow contracture release open surgical approach *revision ulnar nerve transposition performed by a different surgeon Lateral Collateral Ligament (LCL); Medial Collateral Ligament (MCL); Open Reduction Internal Fixation (ORIF)

	Incision	Medial approach	Lateral approach	Ulnar nerve transposition	Heterotopic bone excision	Other
1	Posterior	x	x	x	x	
2	Posterior	x		x	x	
3	Posterior	x	x	x	x	Removal of hardware
4	Posterior	x	x	x	x	
5	Posterior	x	x	x		Removal of loose bodies
6	Posterior		x			LCL repair; radial head replacement
7	Medial	x	x	x		Removal of hardware; radial head replacement; LCL reconstruction; MCL reconstruction; coronoid reconstruction
8	Posterior	x	x	x	x	
9	Posterior		x		x	Removal of hardware
10	Posterior	x	x	x	x	Radial nerve release; triceps repair
11	Posterior	x		x*		
12	Posterior	x		x		Removal of loose bodies
13	Posterior		x	x		Removal of hardware
14	Posterior	x	x	x		ORIF capitellar trochlear distal humerus malunion; LCL repair
15	Posterior		x			Revision ORIF ulnar segmental fracture; open reduction radial capitellar joint; LCL repair

The mechanisms of initial injury resulting in elbow contracture for all 19 patients included non-traumatic (osteoarthritis) and post-traumatic (motor vehicle accidents, falls, bicycling accidents) mechanisms.

The results comparing preoperative ROM to research visit ROM for the 19 patients are summarized in Table 3.

**Table 3:** Preoperative ROM vs. research visit ROM Range of Motion (ROM)

	Preoperative ROM			Research visit ROM			
	Average	SD	Range	Average	SD	Range	p Value
Flexion	97.6	32.3	125	131.3	14.4	65	0.0003
Extension	27.1	22.0	85	24.7	22.7	75	0.6886
Flexion-extension arc	72.1	42.4	115	106.6	30.1	140	0.0022
Pronation	71.8	24.6	90	73.2	14.5	50	0.7720
Supination	60.0	35.0	90	68.2	24.2	90	0.2271
Pronation-supination arc	131.8	53.2	180	141.3	33.7	135	0.3306

**Figure 1:**
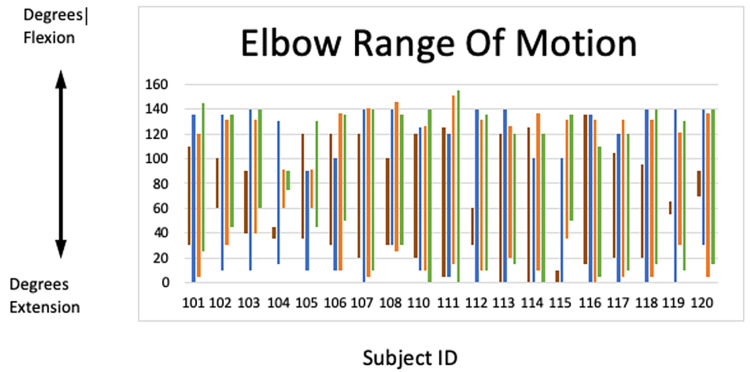
Elbow range of motion Red: Preoperative ROM; Blue: Immediate postoperative ROM (obtained from the operative report or first postoperative clinic visit when data are not available in the operative report); Orange: Final clinic visit ROM; Green: Research visit ROM Range of Motion (ROM)

The difference between preoperative flexion and research visit flexion measurements was statistically significant (p<0.001), as was the difference between preoperative flexion-extension arc and research visit flexion-extension arc (p<0.01). There was no significant difference between preop extension and research visit extension measurements (p<0.6886) and no significant differences between preoperative and research visit pronation (p=0.772), supination (p=0.2271), and the pronation-supination arc (p=0.3306). The mean change in the flexion-extension arc between the preoperative visit and the research visit was 36.4° (SD ± 39.7, range: min. -15°, max. 110°), and the mean change in the pronation-supination arc was 9.0° (SD ± 42.41, range: min. -45°, max. 130°). The flexion-extension arc at preoperative, immediate postoperative, final postoperative clinic visit, and research visit time points for all 19 patients is individually plotted in Figure 1.

The grip strength mean of the operative side was 30.0 lbs. (SD ± 22.3) and the grip strength mean of the nonoperative side was 35.8 lbs. (SD ± 17.6). The difference between the two means was not significant (p= 0.18).

All patients completed the following clinical outcome questionnaires to assess pain, function, and surgery satisfaction: ASES-e, SST-e, DASH, MEPI, and SF-36. The average scores are reported in Table 4.

**Table 4:** Patient questionnaire scores at research visit American Shoulder and Elbow Surgeons-elbow (ASES-e); Simple Shoulder Test-elbow (SST-e); Disabilities of the Arm and Shoulder (DASH); Mayo Elbow Performance Index (MEPI); Short Form-36 (SF-36)

	Average	SD	Range
ASES-e pain score	38.00	12.33	4-50
ASES-e function score	31.95	3.56	24-36
ASES-e surgery satisfaction score	8.84	1.63	5-10
SST-e score	78.95	27.50	8.3-100
DASH score	15.57	19.36	0-73.3
MEPI score	86.32	17.16	40-100
SF 36 score	73.95	24.79	15-100

Each patient completed the Hand Pain and Numbness Diagram (Appendix A). Five patients had ulnar nerve symptoms of pain, paresthesia, and/or decreased sensation. Of these five patients, four had ulnar nerve transposition at the time of elbow contracture release surgery. A total of 12 of the 19 patients underwent an ulnar nerve transposition. The remainder of the physical exam sensory findings are reported in Table 5.

**Table 5:** Research visit physical exam sensory measurements *The monofilament test was divided into normal (2.83 monofilament size) and abnormal (>2.83 monofilament size).

	Operative side	Nonoperative side
Tinel's sign		
Positive	5	2
Negative	14	17
Cubital Tunnel Stretch test		
Positive	7	4
Negative	12	15
Ulnar nerve subluxation		
Positive	0	0
Negative	19	19
2-point discrimination test		
Index		
Normal (0-5mm)	17	17
Fair (6-10mm)	1	2
Poor (11-15mm)	1	0
Small		
Normal (0-5mm)	14	17
Fair (6-10mm)	4	2
Poor (11-15mm)	1	0
Monofilament test*		
Median Nerve Volar Thumb		
Normal (2.83)	14	
Abnormal (> 2.83)	5	
Median Nerve Distal Index		
Normal (2.83)	13	
Abnormal (>2.83)	6	
Median Nerve Proximal Index		
Normal (2.83)	17	
Abnormal (>2.83)	2	
Ulnar Nerve Distal Small		
Normal (2.83)	13	
Abnormal (> 2.83)	6	
Ulnar Nerve Proximal Small		
Normal (2.83)	11	
Abnormal (>2.83)	8	
Ulnar Nerve Palm		
Normal (2.83)	12	
Abnormal (>2.83)	7	

Of the 19 patients enrolled, 16 completed the additional investigator questionnaire (Appendix B). All 16 patients replied yes to the question “Are you happy with the decision to have the contracture release?” and “If given the choice, would you have the surgery again?” Regarding “what patients reported liking most about the post-surgical outcome,” eight patients stated increased ROM, eight patients stated reduced pain, and five mentioned better ability to perform ADLs. Patients listed the scar, post-traumatic arthritis, and inability to lift/push heavy objects as things they least like regarding postsurgical outcomes. All 16 patients stated they are able to complete all ADLs without difficulty. Fifteen patients felt their elbow function is better than the day before surgery, one patient felt it was worse. Fourteen patients reported having pain in addition to decreased ROM prior to surgery and all fourteen reported their level of pain was reduced after surgery. Of those 14 patients, six patients reported 100% pain reduction, five patients reported 50%-90% pain reduction, two patients reported 25%-50% pain reduction, and one patient reported 0%-25% pain reduction.

## Discussion

Prior studies have reported satisfactory outcomes following surgical elbow contracture release [11-14,29- 30]. However, few studies assess long-term outcomes and patient-reported satisfaction. The primary goals of treatment for a stiff elbow are to restore a functional arc of motion, decrease or eliminate pain, and provide stability [9]. In the current study, we found maintenance in flexion and the flexion-extension arc with long-term follow-up, averaging 58.9 months (SD± 39.8, range 22-117) at the research visit. Our data would support that a patient self-guided postoperative therapy protocol with an outpatient therapy program was effective in maintaining range of motion. Our clinical outcome data showed most patients are able to comfortably complete ADLs, have minimal pain, and are highly satisfied with their long-term functional outcome of surgery and the decision to have elbow contracture release surgery. Our patient population reported excellent long-term outcomes regarding a decrease in pain. The average scores for the patient-reported outcome questionnaires demonstrate decreased pain and high satisfaction in regard to surgical outcomes and the responses to the investigator-written questionnaire supported increased ROM, ability to perform ADLs, and decreased pain for the majority of patients enrolled in this study.

In regard to restoring motion, Sojbjerg suggested that elbow flexion is more important than extension in order to perform ADLs [8]. More recently, increased functional ROM degrees have been suggested to adequately complete contemporary activities of daily living, as holding a cell phone to the ear may require increased flexion, and typing on a computer requires increased pronation [3,5-7]. Our clinical outcome data would support that elbow contracture release surgery maintains increased ROM arc and flexion, decreased pain, and functionality long-term. The SST clinical outcome score assesses the patient’s ability to perform ADLs such as performing work duties, dressing, bathing, lifting, carrying, and throwing, with a score of 0 equating to unable to perform and 100 being able to easily perform ADLs. Our cohort’s average SST score was 78.95, indicating that most patients are able to successfully perform ADLs. The DASH clinical outcome scores were utilized to assess the degree of difficulty in performing differing physical activities with the affected arm, the severity of pain symptoms, activity-related pain, tingling, weakness, and stiffness, as well as the affected elbow’s impact on social activities, work, sleep, and self-image. Our cohort’s average DASH score of 15.57 is rather low, indicating close to no disability when using their surgically released elbow. The MEPI measures elbow function across four domains: pain, stability, motion, and daily functional tasks. Our cohort’s average MEPI score of 86.32 is considered a “good” score categorization. This patient cohort consistently demonstrated favorable clinical outcome scores in terms of ability, pain, and satisfaction. In doing so, we are able to counsel patients that they are likely to return to full ability and pain-free ADLs following elbow contracture release surgery with sustained improvement in their elbow flexion.

There are reports of the use of CPM devices in the postoperative period following surgical elbow contracture release [11-15]. Some studies have suggested CPM use following surgical elbow contracture release demonstrates advantageous effects in restoring flexion and extension ROM when compared to no-CPM use and extension splinting [14]. A retrospective study by Aldridge et al. reported an improvement in the total arc of motion of 45° ± 3° in patients treated with CPM compared to 26° ± 5° in those not treated postoperatively with CPM. Gates et al. demonstrated a significant difference in the increased mean total arc of motion between the CPM cohort and the non-CPM cohort of 27° and 48°, respectively [14]. However, an important limitation of this study by Gates et al. is that there was no standardization between the CPM and no-CPM cohort, as prior to contracture release the no-CPM cohort had substantially stiffer elbows than the CPM cohort [14]. Cohen and Hastings reported a mean arc of motion increase from 74° to 129° in 22 patients following surgical elbow contracture release and postoperative CPM use [13]. In contrast, Lindenhovius et al. demonstrated that patients, evaluated between seven months and 56 months postoperatively, who did not use CPM in their postoperative rehabilitation protocol had similar results in terms of ROM change and restoration when compared to the literature of patients who did use CPM [16]. The improvement in ROM measurements in our study support sustained ROM in the flexion-extension arc at the long-term follow-up, ranging from 22 months to 58 months, without the use of CPM. Our study demonstrates a statistically significant improvement in flexion ROM, leading the authors to suspect that extension ROM may be limited over time due to the anterior capsule of the elbow joint getting tight over time and likely scarring down to the anterior humerus, as most elbow resting positions in some degree of flexion and terminal extension may therefore be lost. Ultimately, our study demonstrates patients do well long-term with a self-directed/home therapy program guided by OT/PT without the use of CPM.

Given the nature of the ulnar nerve’s close anatomical proximity and involvement in elbow contracture release, it is important to assess residual nerve dysfunction at the long-term follow-up. On physical exam sensory testing, most patients in our cohort reported minimal sensory dysfunction (Table 5). These findings were also supported by our cohort’s low DASH score average (15.57). The Hand Pain and Numbness Diagram also demonstrated five patients to have sensory symptoms in the ulnar nerve distribution, and four of these patients did have an ulnar nerve transposition at the time of elbow contracture release surgery. This is important when counseling patients regarding residual nerve dysfunction and symptoms when the ulnar nerve is transposed. However, we had very limited preoperative sensory testing data to appropriately determine if sensory function improved, worsened, or stayed the same following surgery. Strength in the affected arm at long-term follow-up compared favorably to the unaffected arm. The grip strength mean of the operative side was 30.05 lbs. (SD ± 22.3) and the grip strength mean of the non-operative side was 35.8 lbs. (SD ± 17.6). While we cannot comment on the maintenance of strength from surgery to long-term follow-up, we can counsel patients that their grip strength in the surgical hand will return to similar to the non-operative side.

We recognize this study has several limitations. This is a case series and, therefore, a small sample size. Second, while this study reported the long-term outcome of surgical elbow contracture release in a patient group, we did not have a comparison control group to demonstrate the direct effects of the use of CPM versus no CPM and, therefore, future research should focus on the direct comparison between these rehabilitation protocols. Third, preoperative ROM measurements were gathered via retrospective chart review, which leaves room for the introduction of bias and human error, however, a goniometer is routinely used in the clinic to measure ROM. Lastly, we recognize the test measurements performed at a comprehensive research visit were not also available in the preoperative setting for our patient cohort due to the study’s design and the overall study design and methods can be improved upon in the future.

## Conclusions

Elbow contracture release surgery demonstrates improvement and maintenance in flexion and flexion-extension arc with long-term follow-up. The long-term postoperative outcome for surgical elbow contracture release followed by a self-directed, OT/PT-guided physical therapy program without CPM is favorable. Furthermore, patients were satisfied with long-term postoperative outcomes related to performing ADLs, decreased pain, and the decision to have the surgery.

## Figures and Tables

**Figure 2:**
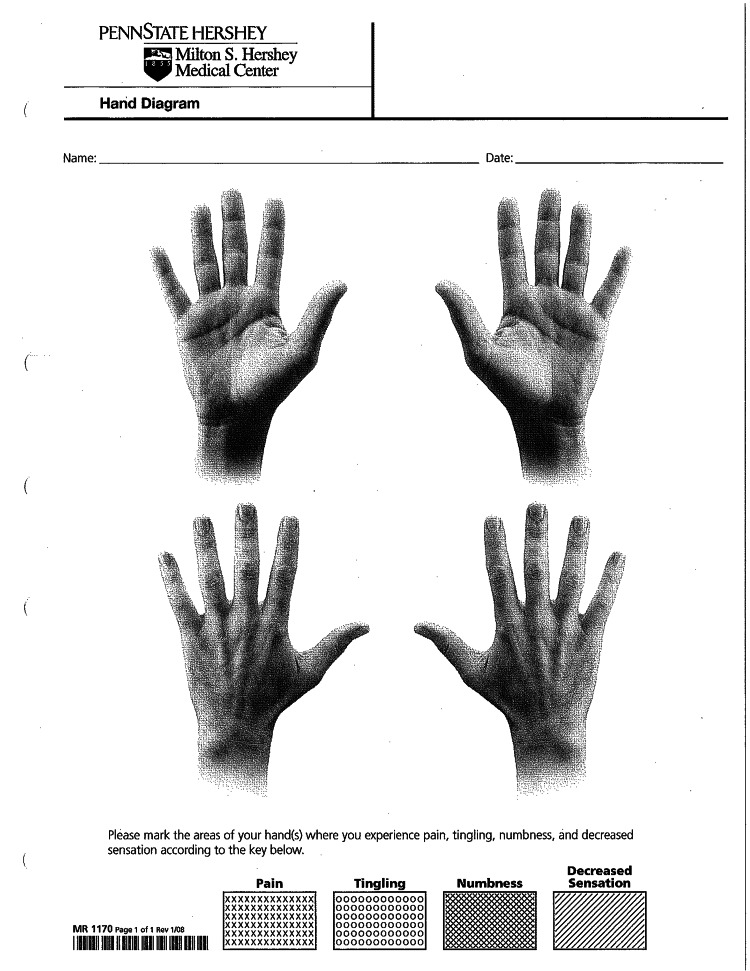
Hand Pain and Numbness Diagram

## Appendices

Appendix A

Hand Pain and Numbness Diagram

Appendix B

Investigator Written Questionnaire

Please take the time to read and answer each question carefully.

1.)   On the line below, please indicate your level of pain prior to the contracture release of your elbow.

      No Pain |______________________________________________________| Worst Possible Pain

2.) Do you currently have elbow pain?            Yes______        No_______

a.)  If Yes, please indicate your pain level on the line below.

No Pain |______________________________________________________| Worst Possible Pain

b.) If No, how long after the contracture release did it take for your elbow pain to go away?

      _____________________________________________________________________________________________________

3.)  Are you happy with the decision to have the contracture release?            Yes______        No_______

4.)  Did anything influence your decision to have the contracture release?    Yes______        No_______

a.) If Yes, please explain _____________________________________________________________________________

5.) If given the choice, would you have the surgery again? Yes______ No______

6.) Have you had any additional surgeries since having the contracture release? Yes______ No______

            a.) If Yes, what was done? __________________________________________________________________________

7.) What do you like most as a result of the contracture release? _______________________________________ ________________________________________________________________________________________________________

8.) What do you like least as a result of the contracture release? _______________________________________  ______________________________________________________________________________________________________

9.) What activities of daily living are you still able to do without much difficulty? (ie: brushing teeth, brushing hair, washing dishes, bathing, personal hygiene) _______________________________________________________________________________________________________________________________________________

10.) What activities of daily living do you have trouble performing? (ie: brushing teeth, brushing hair, washing dishes, bathing, personal hygiene)_________________________________________________________________________________________________________________________________________________________________

11.) Is your elbow function worse / the same / better than the day before your surgery? Please circle one.

12.) What advice would you give someone considering a contracture release?_____________________________________________________________________________________________________________________________________

13.) After the contracture release, was your level of pain reduced? Yes______ No______

            a.) If Yes, please rate how much your pain was reduced by having the contracture release.

            Slightly (0-25%)      Moderately (25-50%)        Significantly (50-90%)      No Pain

14.) Please feel free to provide additional comments: ____________________________________________________

Thank you for completing this survey.
